# Comparison of variables measured with a Scheimpflug device for evaluation of progression and detection of keratoconus

**DOI:** 10.1038/s41598-020-76020-6

**Published:** 2020-11-09

**Authors:** Sophie Neuhann, Anna Schuh, Daniel Krause, Raffael Liegl, Valerie Schmelter, Thomas Kreutzer, Wolfgang J. Mayer, Thomas Kohnen, Siegfried Priglinger, Mehdi Shajari

**Affiliations:** 1 Department of Ophthalmology, Ludwig-Maximilians University, Munich, Germany; 2 Department of Ophthalmology, Goethe University, Frankfurt, Germany

**Keywords:** Corneal diseases, Vision disorders, Preventive medicine

## Abstract

Keratoconus is a progressive ectatic corneal disorder, which can result in severe visual impairment. The new ABCD keratoconus classification system allows differentiated description of the disease. Aim of the study was to evaluate the components of this novel staging system (ARC, PRC, thinnest pachymetry) as well as topometric indices, deviation of normality indices, and other parameters in terms of repeatability and reliability. 317 eyes with keratoconus were examined twice with a Scheimpflug device (Pentacam, Oculus). Bland Altman analysis and intraclass correlations were carried out to evaluate the parameters repeatability and reliability. Apart from IHA (ICC = 0.730), all parameters showed excellent reliability (ICC > 0.900). ARC, PRC, thinnest pachymetry, Kmax, CKI, KI, Rmin, and Progression Avg were the best repeatable parameters with relative repeatability values < 2.5%. Other parameters performing well in terms of repeatability were IHD, ISV, IVA, and final D (RR < 13%). Regression analysis revealed consistently high repeatability along all stages of keratoconus for PRC, thinnest pachymetry, and CKI. All parameters of the ABCD staging system showed excellent reliability and repeatability, PRC and thinnest pachymetry even at all stages of keratoconus and can consequently be reliably used in the determination of keratoconus progression.

## Introduction

Keratoconus is by definition a progressive ectatic corneal degenerative disorder characterised by the protrusion and stromal thinning of the cornea, which can result in severe visual impairment^1,2^. The speed of progression varies, however, widely – from rapid to extremely slow progression.

In the past, the speed of progression could only be observed, but not influenced.

However, with the development of modern therapeutic possibilities to slow or even halt progression of such ectasia, namely collagen crosslinking^3^, the determination of the speed of progression becomes of great importance for establishing the indication for such treatment in view with its associated risks^4^.

The current standard for the detection and progression evaluation of keratoconus is corneal topography/tomography with the Scheimpflug technique, e.g. Pentacam, creating three-dimensional imaging of the entire anterior eye segment and providing a wide array of information through automated measurements and analyses.

The Pentacam’s technology has been developed from measuring standard parameters, such as keratometry, pachymetry and corneal elevation to allow the calculation of various corneal indices including anterior-surface topometric indices (IHA, IHD, IVA, ISV, KI, CKI) as well as deviation from normality indices (D-index).

A new classification system has since been proposed, which includes the anterior and posterior radii of curvature, as well as the corneal thickness at the thinnest point and best distance visual acuity. For each of these parameters five stages have been defined, which allows a differentiated description of the individual’s disease^5^. Furthermore, it emphasises the importance of posterior corneal changes, which may occur even before changes in the anterior surface and are therefore substantial in detecting early keratoconus progression^6^.

These parameters are displayed in the progression display included in the Pentacam software. Their value for progression detection depends critically on the accuracy and repeatability of measurements in order to distinguish actual progression from mere errors, inaccuracy and/or variability in measurement.

The purpose of this study was to determine, whether the new grading system and its components, namely the anterior and posterior radii of curvature as well as thinnest pachymetry can precisely be measured in repeated exams, as to reliably document potential progression.

Furthermore, other parameters have been re-examined in a large sample for their value in the evaluation of keratoconus.

## Material and methods

A total of 317 eyes of 185 patients were examined at the Department of Ophthalmology of the Ludwig-Maximilians-University in Munich, Germany, using the Scheimpflug method (Pentacam, Oculus, Wetzlar). To examine the repeatability of various measures, each eye was measured twice within one day to ensure that any changes in values could only be attributed to the device’s measurement variability and not actual progression of the patients’ keratoconus.

To be included in this study, patients had to be diagnosed with manifest keratoconus in one or both eyes. Patients who had other ectatic diseases, corneal scarring, infections or had undergone previous corneal surgeries were excluded from the study.

The measured parameters included the steepest radius of curvature (Kmax), the thinnest pachymetry (Pachymin), distance from apex to thinnest location (Distapex), back (ELEB) and front (ELEF) elevation, Ambrósio relational thickness maximum (ARTmax), anterior (ARC) and posterior (PRC) radius of curvature 3 mm from the thinnest point, as well as the simplified staging indices for ARC (A), PRC (B), and pachymetry (C) were analysed. Furthermore, the keratoconus index (KI), central keratoconic index (CKI), index of vertical asymetry (IVA), index of height asymmetry (IHA) and decentration (IHD), index of surface variance (ISV) and the minimum radius of sagittal curvature (RMI) were evaluated.

Also, the Belin/Ambrósio Enhanced Ectasia Display Software was used to generate the D-index, consisting of the five subgroups Df (deviation of front surface elevation difference), Db (deviation of back surface elevation difference), Dp (deviation of pachymetric progression), Dt (deviation of thinnest point), and Da (deviation of ARTMax/Ambrósio relational thickness maximum).

Only images rated as “ok”by the system were used for the analysis.

The study protocol was reviewed and approved by the Institutional Review Board of Ludwig-Maximilians-University Munich (Ethikkommission LMU). Written informed consent was waived by the Ethics Committee that approved this study’s protocol. The tenets of the Declaration of Helsinki were followed throughout the study.

### Data and statistical analysis

Statistical analysis was performed using Stata 14 (Statacorp) and SPSS 25 (IBM).

For the evaluation of repeatability, Bland Altman analysis^7^ showing the mean difference between the times of measurements and the standard deviations, the coefficient of reliability (COR), and the 95% limits of agreement (LOA) were calculated. Limits of agreement are defined as the mean difference ± COR, which is 1.96 × the standard deviation of the mean difference. It is the range estimating the limits, within which 95% of differences between measurements lie.

Further, intraclass correlations (ICC) were calculated for all parameters as a measurement of intra-rater reliability. ICC values of 0.900 are considered as excellent reliability, values between 0.750 and 0.900 as good reliability, values between 0.500 and 0.750 as moderate reliability, and values below 0.500 as poor reliability^8^. Relative repeatability (RR), the percentual ratio of COR to the total mean value of the parameter, is used to facilitate the interpretation of the results. Smaller values indicate a smaller deviation from the mean difference relative to the total mean values of the parameter and therefore more accurate measurements.

## Results

A total of 185 patients, aged on average 32.25 years (range 7–71 years) were examined.

Table 1 shows total means and SD, the mean differences between measurements and its SD, COR, lower (L LOA) and upper (U LOA) limits of agreement, as well as ICC and RR for all measured parameters.

**Table 1:** Bland Altman Alalysis of repeated measurments. N = number of eyes, COR = coefficient of repeatability, L LOA = Lower limit of agreement, U LOA = Upper limit of agreement, ICC = Intraclass correlation, RR = Relative repeatability (COR/Mean), ARC = anterior radius of curvature, A = staging index for ARC, PRC = posterior radius of curvature, B = staging index for PRC, Pachymin = the thinnest pachymetry, C = staging index for pachymetry, Kmax = steepest radius of curvature, Distapex = distance from apex to thinnest location, Progression Avg = index of average progression, ARTmax = Ambrósio relational thickness maximum, DF = deviation of front surface elevation difference, DB = deviation of back surface elevation difference, D* p* = deviation of pachymetric progression, DT = deviation of thinnest point, DA = deviation of ARTMax/Ambrósio relational thickness maximum, D = D-index , ISV = index of surface variance, IVA = index of vertical asymmetry, KI = keratoconus index, CKI = central keratoconic index, IHA = index of height asymmetry, IHD = index of height decentration, RMI = minimum radius of sagittal curvature.

	N	Mean	SD	Mean Difference	SD	COR	L LOA	U LOA	ICC	RR (%)
ARC	317	7.003	0.598	0.003	0.054	0.106	− 0.103	0.110	0.996	1.514
A	317	1.689	1.292	− 0.003	0.122	0.238	− 0.242	0.235	0.996	14.091
PRC	317	5.311	0.613	0.004	0.067	0.131	− 0.127	0.134	0.994	2.467
B	317	2.507	1.388	− 0.004	0.183	0.358	− 0.362	0.354	0.991	14.280
Pachymin	317	473.730	41.188	0.341	5.748	11.266	− 10.926	11.607	0.990	2.378
C	317	1.424	0.783	− 0.008	0.121	0.238	− 0.245	0.230	0.988	16.713
Kmax	316	52.447	6.068	− 0.004	0.472	0.926	− 0.930	0.922	0.997	1.766
Front Elevation	317	16.820	10.938	− 0.060	2.108	4.131	− 4.191	4.071	0.982	24.560
Back Elevation	317	40.880	22.386	0.079	4.052	7.942	− 7.864	8.021	0.984	19.428
Distapex	317	0.867	0.309	0.003	0.139	0.273	− 0.270	0.275	0.899	31.488
Progression Avg	315	1.836	0.604	− 0.003	0.118	0.023	− 0.233	0.228	0.981	1.253
Artmax	316	198.910	91.754	0.367	22.571	44.240	− 43.873	44.607	0.970	22.241
DF	317	7.291	5.878	0.021	1.109	2.174	− 2.153	2.196	0.982	29.818
DB	317	6.108	4.993	0.001	1.000	1.960	− 1.959	1.961	0.980	32.089
DP	316	6.249	3.966	− 0.036	0.780	1.529	− 1.565	1.493	0.981	24.468
DT	317	2.222	1.591	− 0.011	0.221	0.434	− 0.445	0.423	0.990	19.531
DA	317	2.641	0.837	− 0.004	0.206	0.404	− 0.408	0.400	0.970	15.297
D	317	6.725	3.642	0.001	0.413	0.810	− 0.809	0.812	0.994	12.908
ISV	316	69.918	36.673	− 0.153	2.539	4.977	− 5.128	4.825	0.998	7.118
IVA	317	0.780	0.458	− 0.002	0.042	0.082	− 0.084	0.080	0.996	10.513
KI	317	1.187	0.125	0.001	0.015	0.029	− 0.029	0.030	0.993	2.443
CKI	316	1.034	0.074	0.000	0.005	0.011	− 0.011	0.011	0.997	1.064
IHA	317	28.549	23.988	− 1.205	17.597	34.489	− 35.694	33.285	0.731	120.806
IHD	315	0.105	0.068	0.000	0.006	0.011	− 0.011	0.011	0.997	10.476
RMI	315	6.516	0.707	0.002	0.049	0.096	− 0.094	0.098	0.998	1.473

With the exception of Distapex (ICC = 0.899) and IHA (ICC = 0.731), all parameters showed excellent ICC values (> 0.97).

ARC, PRC, Pachymin, Kmax, Progression Average, KI, CKI, and RMI (RR < 2,5%), as well as ISV, IVA, IHD and final D (RR < 13%) showed the lowest relative repeatability values. With the exception of IHA (RR = 120,8%) all other RR values ranged between 14 and 32%.

**Figure 1:**
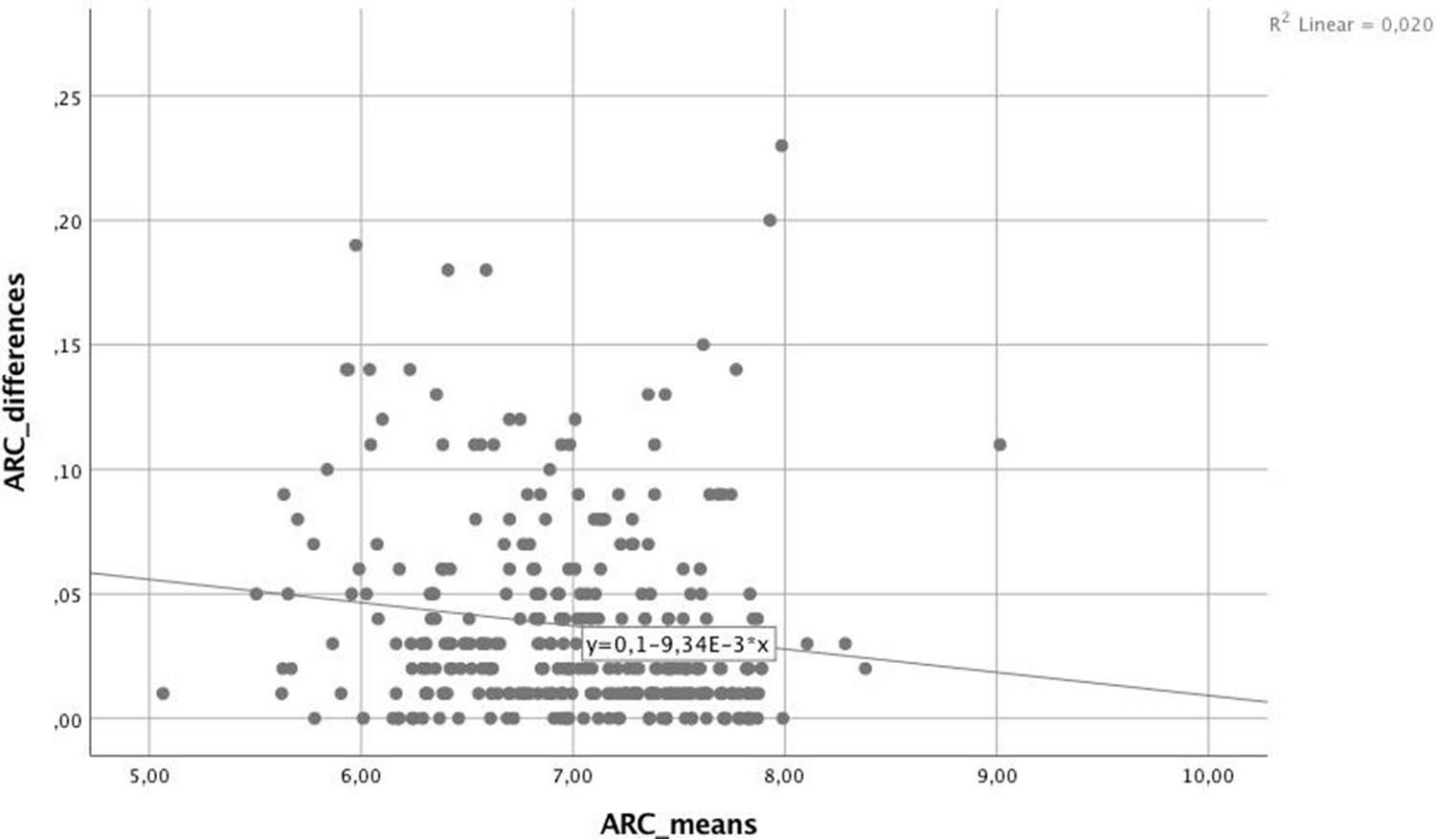
For the anterior radial curvature (ARC) a negative trend between measurements was found for higher curvature values.

**Figure 2:**
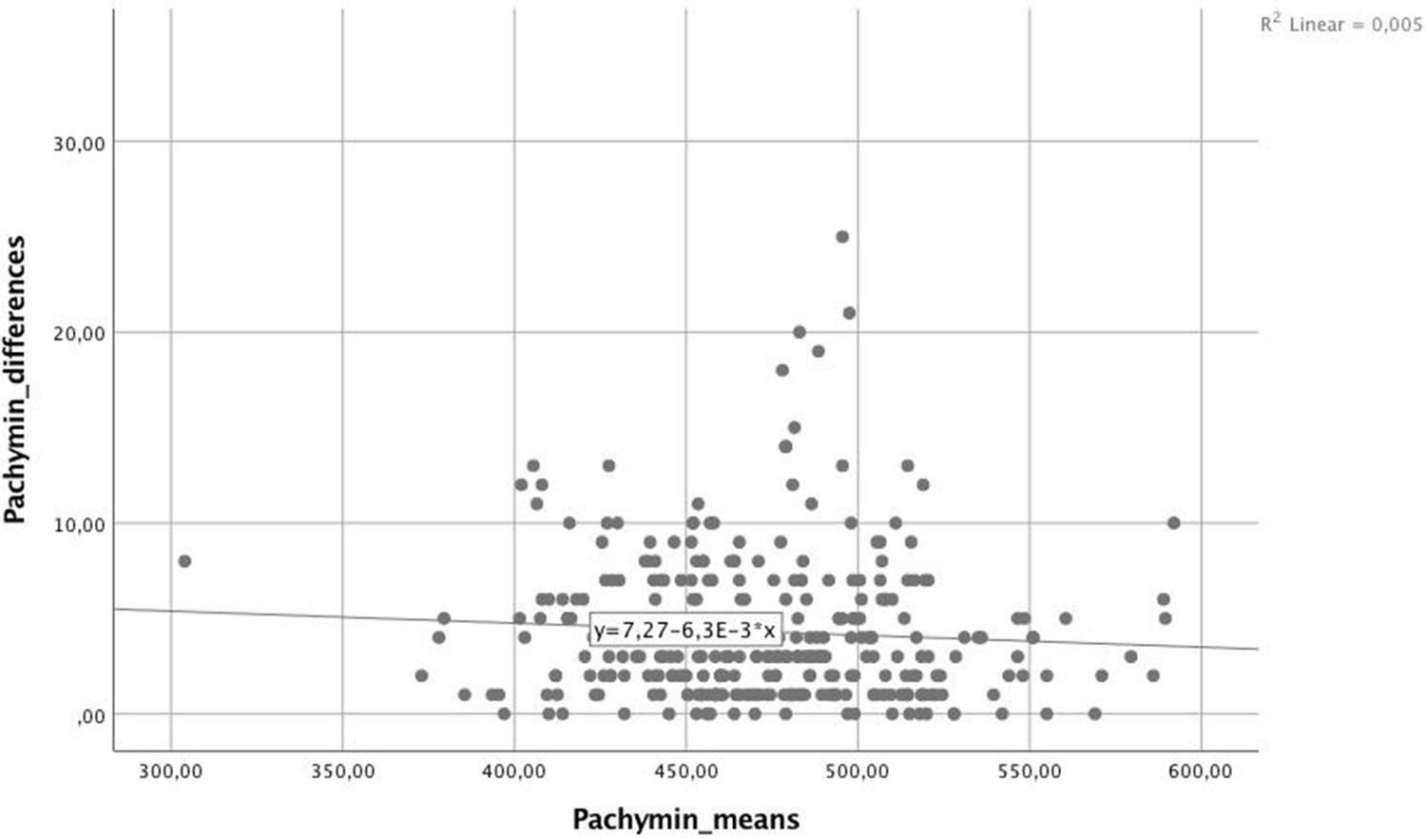
For Corneal thickness at the thinnest point (Pachymin) there was no significant difference between measurements visible at higher values.

For the parameters with the lowest RR (< 2.5%), linear regression analysis was carried out to identify possible trends regarding the absolute differences between measurements relative to the mean values of each parameter, shown in Table 2. The linear regression analysis yielded significant values (*p* < 0.05) for ARC (Fig. 1), Kmax, Progression Avg, KI, and Rmin, indicating significantly increasing absolute differences in measurements at higher stages of keratoconus. Such a trend could not be found for PRC (*p* = 0.230), Pachymin (*p* = 0.892; Fig. 2), and CKI (*p* = 0.306).

**Table 2:** Regression analysis. r^2^ = coefficient of determination, ARC = anterior radius of curvature, PRC = posterior radius of curvature, Pachymin = the thinnest pachymetry, Kmax = steepest radius of curvature, Progression Avg = index of average progression, KI = keratoconus index, CKI = central keratoconic index, Rmin = smallest radius index.

	Coefficient	r^2^	*p*
ARC	− 0.009	0.02	0.012
PRC	0.001	0	0.892
Pachymin	− 0.006	0.005	0.230
Kmax	0.017	0.082	0.000
Progression Avg	0.065	0.191	0.000
KI	0.019	0.037	0.001
CKI	0.004	0.003	0.306
Rmin	− 0.01	0.034	0.001

## Discussion

The Pentacam technology allows for a detailed display of the corneal shape and its abnormalities to detect early changes. In the present study, those parameters, that are commonly used for defining keratoconus, and its progression over time, as well as topometric indices and deviation from normality indices, were assessed for measurement repeatability and reliability in a large sample of keratoconic eyes. Since progression of the disease is the deciding factor in the indication of treatment it needs to be clearly distinguishable from “forme fruste” keratoconus or a mere variance in measurements at the earliest possible point in time, the purpose was to find best repeatable and reliable determinants in the diagnosis and progression evaluation of corneal ectasia.

For the parameters forming the ABCD grading system, ARC, PRC, and minimum Pachymetry, 95% of differences between measurements were very close to zero, as represented by excellent intraclass correlations (> 0.990) and uniformly low relative repeatability values (< 2,5%), indicating that these parameters are among the best repeatable and reliable parameters evaluated in this study. These results go in line with a recent study by Duncan et al.^9^, which obtained small confidence intervals in repeated measurements for all three parameters, suggesting their suitability as progression determinants. Regression analysis showed a significant increase in the size of the measuring differences – caused by measurement variability—with increasing stages of keratoconus for ARC (r^2^ = 0.02, *p* = 0.012), suggesting that measurements do become less accurate as the corneal ectasia progresses. Although 95% of differences were within relatively narrow limits (L LOA = -0.103 mm, U LOA = 0.110 mm), this decreasing accuracy needs to be taken into account when diagnosing progression in more advanced keratoconus. For PRC and minimum Pachymetry, measurements were found to be equally accurate across all values (*p* > 0.05). Together with findings by a recent study by Kosekahya et al.^10^ that the yearly change rate of the thinnest pachymetry was found to significantly differ between nonprogressive and progressive keratoconus, thinnest pachymetry can be suggested as a potentially excellent tool in the progression evaluation.

Other parameters that performed excellently in terms of repeatability (RR < 2.5%) were maximum K, Progression Avg, KI, CKI, and Rmin. To see, whether this level of repeatability can be found across all stages of keratoconus, linear regression analysis was carried out to identify possible trends, as studies have shown that accuracy tends to decrease with progression^11^. While measuring Kmax, Progression Avg, KI, and Rmin became less accurate at more progressed keratoconus, this trend could not be observed for CKI.

With the exception of IHA, all topometric indices performed well in terms of repeatability and reliability, with KI, CKI, and RMI yielding the best results (RR < 2.5%).

IHA was found to perform worst out of all included parameters examined in this study in terms of repeatability (RR = 120.8%) and reliability (ICC = 0.731), similar to results found in previous studies^12,13^. Kosekahya et al.’s study found that measurement variability increased with keratoconus severity for all topometric indices^12^. In this study this trend could not be found for CKI, showing consistently low measurement variability along all stages of keratoconus.

Kanelloupolos et al.^13^ suggested ISV and IHD as good indicators of progression. Along with IVA these parameters ranged among the most accurate (RR between 7 and 10%) in repeated measurements in this study.

Out of all deviation from normality indices, final D showed the best repeatability (RR = 12.91%) as well as excellent reliability (ICC = 0.994). While total D has been recommended as a valuable parameter in the detection^14,15^ and evaluation of ectatic progression in previous studies, especially in early stages of keratoconus^16^, in this study, while performing quite well, it did overall rank lower than other measured parameters in terms of accuracy in repeated exams.

Maximum K is commonly used to diagnose and evaluate the rate of progression in keratoconic eyes. With very small limits of agreement in relation to overall values (RR = 1.77%) and excellent intra-rater reliability (ICC = 0.997), the results of this study second those of many previous studies. Nevertheless, further analysis showed a significant decrease in accuracy at higher values of maximum K. This goes in line with a previous study by Flynn et al.^17^, which found limits of agreement more than doubled at Amsler-Krumeich stages above two.

Of course, this study has its limitations. All measurements were performed by the same examiner. The size of measurement variability has been found to vary. Measurements by different examiners might vary more.

Only two measurements were taken in this study. Although this is closer to clinical reality, it has previously been reported that accuracy of measurement increases by taking three measurements^18^.

A potential for statistical bias is that both eyes of the same patient have been included. We decided nevertheless to include both eyes as keratoconus shows in majority of patients due to its asymmetric progression between both eyes a different pattern in each specific eye. We acknowledge that despite this, it can still have caused a statistical bias but we thought that in a keratoconus cohort the risk was rather low due to the many different patterns which might exist between both eyes and the potential benefit of increased power of the study by a higher sample size outweighs this risk.

In conclusion, from this analysis, the most repeatable parameters were ARC, PRC, minimum Pachymetry, Kmax, Progression Avg, KI, CKI and Rmin. Of these, the high repeatability was even independent of the stage of progression, i.e. of the absolute value of the measured parameter for Pachymin, PRC, and CKI. Therefore, these appear to be parameters best suited in the discrimination between measurement variability and true progression, even at low increments. These findings can be taken into account when assessing progression, but keeping in mind that the relative repeatability value is an average between all stages, thus by trend underestimating progression at lower stages and overestimating it at higher stages. The three parameters used for the modern ABCD staging system, ARC, PRC, and thinnest pachymetry were found to be among the most reliable and repeatable in this study and can be recommended to accurately portray the individual’s disease.

The use of these findings as a basis for the decision to indicate treatment options has to be further evaluated in future clinical practice.
